# Resource Heterogeneity Moderates the Biodiversity-Function Relationship in Real World Ecosystems 

**DOI:** 10.1371/journal.pbio.0060122

**Published:** 2008-05-20

**Authors:** Jason M Tylianakis, Tatyana A Rand, Ansgar Kahmen, Alexandra-Maria Klein, Nina Buchmann, Jörg Perner, Teja Tscharntke

**Affiliations:** 1 Agroecology, Georg-August-University, Göttingen, Germany; 2 School of Biological Sciences, University of Canterbury, Christchurch, New Zealand; 3 Center for Stable Isotope Biogeochemistry, Department of Integrative Biology, University of California at Berkeley, United States of America; 4 Department Environmental Sciences, Policy and Management, University of California at Berkeley, California, United States of America; 5 Institute of Plant Sciences, ETH Zurich, Zurich, Switzerland; 6 U.A.S. Umwelt- und Agrarstudien GmbH, Jena, Germany; McGill University, Canada

## Abstract

Numerous recent studies have tested the effects of plant, pollinator, and predator diversity on primary productivity, pollination, and consumption, respectively. Many have shown a positive relationship, particularly in controlled experiments, but variability in results has emphasized the context-dependency of these relationships. Complementary resource use may lead to a positive relationship between diversity and these processes, but only when a diverse array of niches is available to be partitioned among species. Therefore, the slope of the diversity-function relationship may change across differing levels of heterogeneity, but empirical evaluations of this pattern are lacking. Here we examine three important functions/properties in different real world (i.e., nonexperimental) ecosystems: plant biomass in German grasslands, parasitism rates across five habitat types in coastal Ecuador, and coffee pollination in agroforestry systems in Indonesia. We use general linear and structural equation modeling to demonstrate that the effect of diversity on these processes is context dependent, such that the slope of this relationship increases in environments where limiting resources (soil nutrients, host insects, and coffee flowers, respectively) are spatially heterogeneous. These real world patterns, combined with previous experiments, suggest that biodiversity may have its greatest impact on the functioning of diverse, naturally heterogeneous ecosystems.

## Author Summary

The world is currently experiencing a rapid loss of species, prompting investigation into the role of biodiversity on the functioning of ecosystems. Many recent studies have shown that high diversity of plants, pollinators, and predators is related to high plant growth, pollination, and predation, respectively. Many of these studies involved controlled experiments, yet results were highly variable, indicating that the environment may affect the relationship between species diversity and these ecosystem functions. In a heterogeneous environment, different species can occupy different microhabitats, or use different resources. This reduces competition between species, and can mean that diverse assemblages perform their ecosystem functions at elevated rates. Here we examine rates of three important functions in different natural, nonexperimental ecosystems: plant biomass production in German grasslands, parasitism rates across five habitat types in coastal Ecuador, and coffee pollination in agroforestry systems in Indonesia. We demonstrate that the effect of diversity on these processes increases in environments where limiting resources (soil nutrients, host insects, and coffee flowers, respectively) are spatially heterogeneous. These real world patterns, combined with previous experiments suggest that biodiversity may have its greatest impact on the functioning of diverse, naturally heterogeneous ecosystems.

## Abstract 3

In heterogeneous natural habitats, diverse plant, pollinator, and parasitoid communities can better partition resources and grow, pollinate, and consume at the highest rate.

## Introduction

Global biodiversity decline has prompted a recent explosion of experimental studies addressing the relationship between biodiversity and ecosystem functioning (BDEF) [1–4]. Most of this research has focused on the relationship between diversity and productivity in plant communities (e.g., [5,6]), as reduced primary production due to lost biodiversity may have critical consequences for food production, carbon sequestration, and ecosystem functioning. However, impacts of biodiversity change on function may also be trophically mediated by mobile consumers [7–9]. Therefore, a critical new direction in BDEF research addresses predator-prey interactions [10,11], with important implications for biological pest control, such as the effect of natural enemy diversity on rates of prey consumption [12,13]. Pollination has also been examined within a BDEF framework, as loss of pollinator diversity can reduce pollination rates and may threaten crop production [14–16].

Recent meta-analytical syntheses have shown that on average, reductions in species richness result in a decrease in within-trophic-level abundance or biomass production, less complete resource depletion [17], and can negatively affect ecosystem services [18]. However, results of experiments are notoriously variable [19], with many studies also finding negative [20,21] and no effects [22] of diversity on function. An important development in the BDEF literature has thus been the recognition that the shape and direction of the BDEF relationship can depend critically on environmental context [23–28], which may partly account for the variability in observed outcomes across studies. For example, resource enrichment often strengthens the relationship between plant diversity and productivity [25,29,30], and the relationship between natural enemy diversity and prey consumption rates can vary as a function of prey density [31,32], identity [12], or relative abundance in the environment [33]. Finally, the effects of pollinator diversity on pollination depend on the plant's breeding system and the functioning of key pollinator species, which can change in different habitats or landscape contexts offering different resources [34].

**Figure 1:**
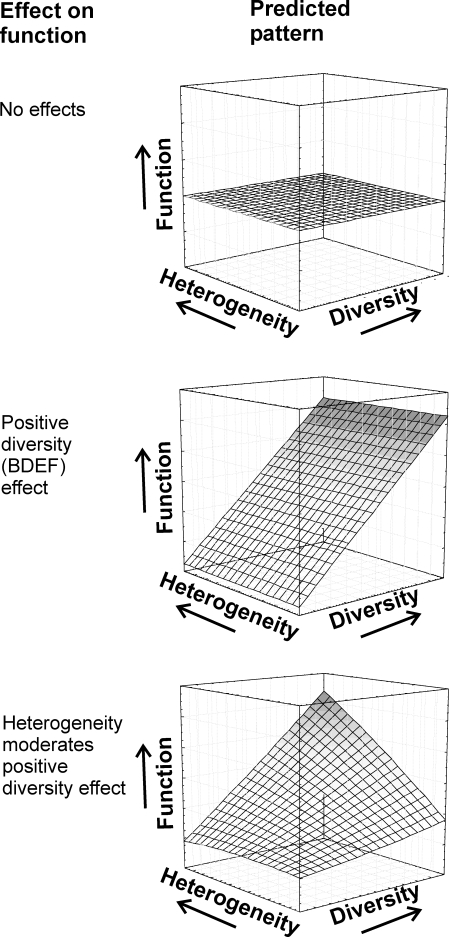
Hypothetical Interactions between Biodiversity and Resource Heterogeneity on Rates of an Ecosystem Function No effect (null hypothesis): neither diversity nor heterogeneity affect functioning. Positive diversity effect: basic diversity-function relationship, with no effect of heterogeneity. Heterogeneity-moderated diversity function: the strength (steepness) of the positive effect of diversity on functioning increases with increasing habitat/resource heterogeneity.

Theoretical studies suggest that spatial heterogeneity in structure or resources can modulate the strength of the diversity-productivity relationship [23], and such heterogeneity has long been known to promote coexistence through resource partitioning among species [35]. As biotope availability (the physical space associated with a species niche) increases along spatial gradients, species may complement each other by occupying slightly different sections of the niche space [27,36]. It may therefore be expected that complementarity effects, whereby resource partitioning among species leads to increased total resource use, would be most strongly expressed in heterogeneous habitats with varied niches, thereby magnifying the effects of biodiversity on functioning (Figure 1). A rare empirical study to examine this possibility [37] found that the influence of species composition (rather than richness) on below ground biomass depended on small-scale heterogeneity in soil resources, suggesting that heterogeneity may modulate the diversity-productivity relationship in plant communities. Surprisingly, we know of no studies that have examined similar issues in a trophic context, despite the fact that natural enemies and pollinators are both known to be strongly influenced by spatial patterns of abundance and diversity of their respective resources [38,39].

The dearth of empirical studies on heterogeneity effects may partly reflect the broader paucity of field studies examining the effects of diversity on productivity or consumption in natural plant or animal assemblages, which are likely to vary in underlying environmental conditions [40] (but see [11,22,41]). This hinders general extrapolation of BDEF results from rigorous experimental studies with low spatial heterogeneity to natural and more complex ecosystems, within which biodiversity loss initially prompted the concern [7,17,40,42]. In fact it is quite likely that the full functional significance of biodiversity will only appear at larger spatial and temporal scales [36,43], and a critical future challenge is to unravel the ecological conditions under which biodiversity has its greatest effect on functional rates [40,42].

Here we show that within-habitat resource heterogeneity strongly moderates the recently demonstrated BDEF relationship in semi-natural and natural ecosystems [11,15,41]. The robustness of this result is underscored by testing diverse functions (belowground standing plant biomass, rates of parasitism, and crop pollination) in different ecosystems (temperate grasslands and a range of tropical habitats on two continents).

## Results

### Plant Biomass

**Figure 2:**
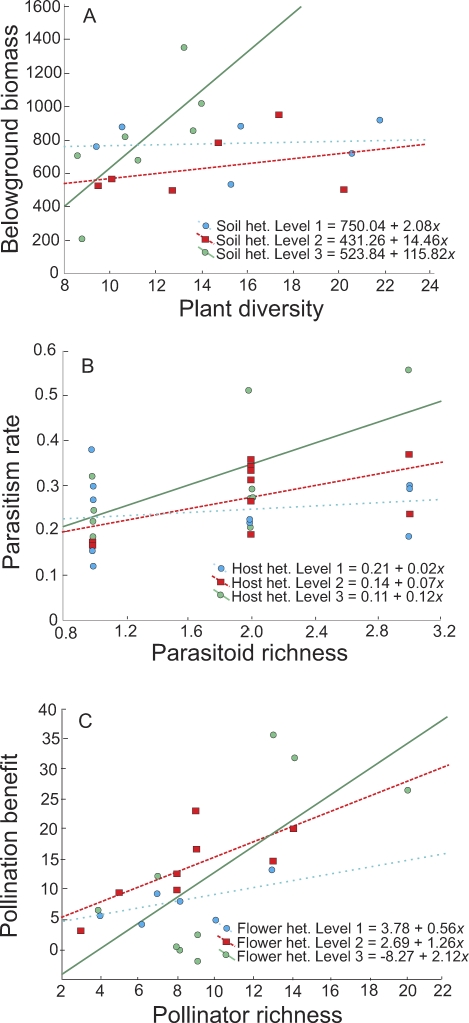
The Slope of the BDEF Relationship Increases with Increasing Resource Heterogeneity In all graphs, the slope of the diversity-function relationship increases with increasing levels of resource heterogeneity. As heterogeneity measures were continuous variables, separation into three categories (1, 2, and 3, representing low, medium, and high heterogeneity, respectively) was necessarily arbitrary. There were an equal number of replicates per heterogeneity level, plus or minus 1 when the number of replicates could not be divided evenly by 3. Equations for the BDEF relationship at each level of heterogeneity are presented with each figure. Both diversity and the interaction between diversity and heterogeneity were significant predictors of the ecosystem function. (A) Relationship between plant diversity (i.e., Shannon-index), soil heterogeneity (Factor 5 from PCA on the CV in soil chemical concentrations), and standing root biomass (dry weight g/m^2^). (B) Relationship between parasitoid species richness, heterogeneity in host (Pseudodynerus sp.) abundance, and rates of parasitism. Heterogeneity in host abundance was calculated as the CV between the nine sampling points per plot. Parasitism rates are the proportion of host larvae parasitized (arcsine square root transformed). (C) Relationship between pollinator species richness, heterogeneity in flower density, and pollination benefit (difference between percent fruit set in open and bagged control treatments). Flower heterogeneity was calculated as the CV in flower abundance between the three experimental coffee shrubs.

In temperate grasslands, plant diversity (i.e., Shannon index; see Methods) was significantly positively correlated with belowground standing biomass (*F*_1,15_ = 8.98*, p* = 0.009, see Text S1A for full parameter values), although no significant predictors remained in the model for aboveground standing biomass (Text S1B). In the minimal adequate regression model for belowground biomass, one of the five estimates of soil heterogeneity, Factor 5 (from a principle component analysis [PCA] to reduce 12 soil heterogeneity variables to five orthogonal factors, see Methods, Table S1), was also significantly positively correlated with belowground standing biomass (*F*_1,15_ = 6.86, *p* = 0.019), and most importantly, the interaction between the effects of plant diversity and soil heterogeneity (Factor 5) was significant (*F*_1,15_ = 8.50, *p* = 0.011) (Figure 2A). This positive interaction was due to the relationship between plant diversity and belowground standing biomass becoming more positive in heterogeneous soils, with the minimum adequate regression model (containing these two variables and their interaction) explaining 41.6% of the variance in belowground standing biomass. It may be argued that two models (one for aboveground, one for belowground biomass), each with 11 predictor variables (plant diversity, five soil factors, and their interactions) may lead to an inflated Type I error rate. Although this risk is often ignored in multifactor models, the probability of obtaining multiple significant effects can be easily calculated using a Bernoulli process [44]: *p* = [*N*!/(*N* − *K*)!*K*!] × α*^K^*(1 − α)*^N^* ^−*K*^, where *N* = the number of “tests” and *K* = the number of tests below α. Therefore, given 22 tested predictors including interactions (11 per model for above- and belowground biomass), the probability of obtaining three predictors significant at *p* < 0.02 (as we did) by chance is a very low *p* = 0.00839, giving us confidence that these effects were not spurious. The congruence between the variables for which we found significant results and previous studies (see below), gives us further confidence that these relationships are not due to chance alone.

**Figure 3:**
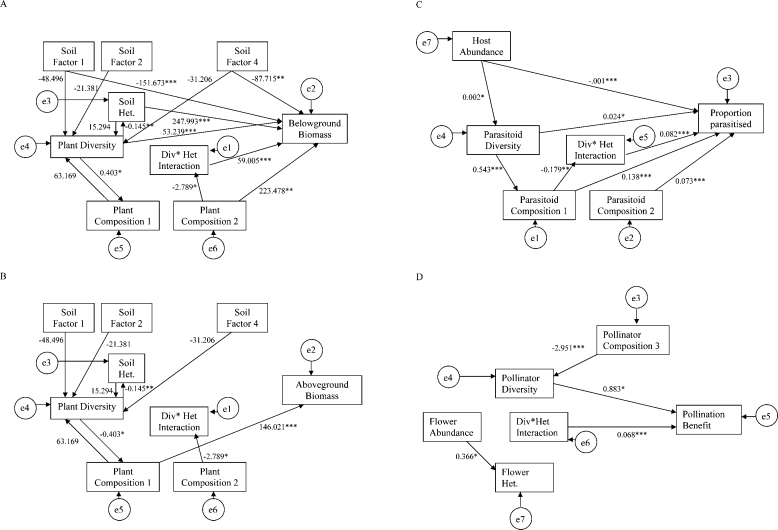
Final Most Parsimonious Structural Equation Models Relating Biodiversity, Resource Heterogeneity, Species Composition, Resource Abundance, and Ecosystem Function Functions shown are: (A) belowground plant biomass, (B) aboveground plant biomass, (C) parasitism rates, and (D) pollination. All paths in the original models (Figures S1–S4) were treated as optional, and were thus able to be removed during model simplification using maximum likelihood estimation. As a final model we selected the model with a subset of the parameters in the initial model that included at least one predictor of function (plant biomass, parasitism, or pollination, respectively), and had the lowest AIC score. Values adjacent to paths indicate unstandardized direct effects, with significance indicated by *, *p* < 0.05; **, *p* < 0.01; ***, *p* < 0.001. For each original and final model, Cmin, AIC, and BCC scores, along with unstandardized and standardized total effects for final models are presented in Text S4.

The significant soil heterogeneity factor (Factor 5) was largely driven by heterogeneity (coefficient of variation [CV]) in NO_3_^−^ concentrations (Pearson's *r* = 0.68), and also by soil carbon and phosphorus concentrations (see factor loadings, Table S2). Importantly, it has been found recently that complementary strategies of inorganic soil nitrogen use among different plant species may lead to a positive biodiversity-productivity relationship in the same N-limited grasslands investigated here [45]. Effects of heterogeneity were not confounded by higher plant diversity in heterogeneous soils, as plant diversity weakly decreased with increasing soil heterogeneity (simple regression: *F*_1,17_ = 5.38, *p* = 0.033, *r* ^2^ = 0.240; Tables S6 and S7). Nutrient availability is known to affect the BDEF relationship in plants [25,28], and it may be argued that nutrient concentrations co-vary with heterogeneity. However, our significant soil heterogeneity factor was not significantly correlated with the concentration of any of the soil variables examined (Figure 3A, Text S2, and Table S3). In the structural equation model (SEM) (Figure 3A), soil heterogeneity had the largest standardized total effect on belowground biomass (Table S7), with the diversity × heterogeneity interaction having the second largest effect. Despite being highly significant (Figure 3A), the size of the effect of plant diversity on biomass was relatively small compared to that of the other parameters (Tables S6 and S7). Although some paths leading to diversity (from three of the soil nutrient availability factors, the heterogeneity factor, and one of the composition axes) remained in the model (Figure 3A), none of these were significant at α = 0.05. Soil nutrient availability and plant species composition significantly affected biomass, although the effects of plant composition axis 1 and soil nutrient availability Factor 2 were entirely indirect, mediated via plant diversity. The SEM for aboveground biomass was unstable, so interpretation of the best fitting model (Figure 3B) must be made with caution. The only significant direct effects in the model were the effects of plant diversity on soil heterogeneity (which had the lowest standardized total effect on soil heterogeneity, Table S9), plant species composition axis 2 on the diversity × heterogeneity interaction, plant diversity on composition axis 1, and the effect of plant composition axis 1 on aboveground biomass. However, the total and standardized total effect of composition on biomass was lower than the effects of each of the soil variables that remained in the model, indicating that availability and heterogeneity of soil nutrients were more important predictors of aboveground biomass than were diversity or composition of the plant community.

### Parasitism

For the ecosystems investigated in coastal Ecuador, we also found that rates of parasitism were significantly higher in plots with high natural enemy diversity compared with low diversity plots (Analysis of Covariance [ANCOVA]: *F*_1,29_ = 15.35, *p* < 0.001, Text S1C), congruent with previous work in our study region using a diverse host guild [11]. Parasitism rates did not vary significantly across habitat types (ANCOVA: *F*_4,29_ = 2.41, *p* = 0.072). Most importantly, however, the strength of the positive effect of parasitoid diversity on parasitism rates increased as within-plot spatial heterogeneity in host abundance increased (ANCOVA, interaction effect: *F*_1,29_ = 5.20, *p* = 0.030) (Figure 2B). This correlation between the BDEF relationship and heterogeneity did not vary significantly across habitat types (ANCOVA, three-way interaction: *F*_4,29_ = 0.99, *p* = 0.427). Host heterogeneity did not affect rates of parasitism directly in the ANCOVA (*F*_129_ = 1.40, *p* = 0.247), instead the effect was mediated indirectly via its interaction with the diversity-parasitism relationship. The overall model explained 55% of the variance in naturally occurring rates of parasitism. The effects of parasitoid diversity were not confounded by effects of host heterogeneity on diversity, as this link was not included in the best fitting structural equation model (Figure 3C; see also Figure 2B). Heterogeneity (CV) in host abundance was also not affected by habitat type, as habitat was not included in the best fitting SEM (Figure 3C). The SEM showed that parasitoid species composition (nonmetric multidimensional scaling [NMDS] axes 1 and 2, see Text S3) had positive effects on parasitism rates that were comparable in magnitude to those of species diversity (Tables S10 and S11), although diversity did have a strong effect on composition axis 1. This indicates that the effect of changing parasitoid diversity on parasitism may be at least partly mediated by changes in parasitoid community composition. The diversity × heterogeneity interaction also had a significant effect on parasitism rates (Figure 3C, Tables S10 and S11). Therefore, even when host abundance and parasitoid composition were considered in the model, parasitoid diversity still positively affected parasitism rates, and this effect became stronger when hosts were heterogeneously distributed.

### Pollination

In Indonesia, agroforests with high bee diversity had higher rates of coffee pollination (Multiple regression: *F*_1,20_ = 8.95, *p* = 0.007, Text S1D) [15], and again, the slope of this relationship increased with increasing spatial heterogeneity in the density of coffee flowers within plots (multiple regression, interaction effect: *F*_1,20_ = 9.42, *p* = 0.006), even though flower heterogeneity had no significant main effect on pollination (multiple regression: *F*_1,20_ = 1.02, *p* = 0.325) (Figure 2C, see also [15]). The overall model explained 49.2% of the variance in enhanced fruit set. These results were reiterated by the SEM (Figure 3D), where the largest standardized total effect on pollination benefit was the interaction between pollinator diversity and flower heterogeneity, with the second largest effect attributed to pollinator diversity (Table S13). Pollinator species composition (NMDS axis 3) also had an indirect effect on pollination, mediated through diversity (Tables S12 and S13). Heterogeneity in coffee flowers had no significant direct effects on pollination, although it was affected by floral abundance (Figure 3D, Tables S12 and S13).

## Discussion

In three different systems (temperate grasslands, host-parasitoid assemblages, and coffee pollinators) across three continents, the relationship between diversity and an ecosystem function became steeper in habitats with spatially heterogeneous distributions of an essential resource. This striking convergence across varied systems and functions indicates a highly robust pattern.

Working at large scales with natural diversity gradients has several limitations, so we needed to consider potentially confounding factors [46]. Because spatial heterogeneity can be an important factor driving patterns of diversity, one issue is that the two are often inextricably correlated, complicating attempts to treat them as independent predictors. In addition, previous models have shown that the direction of the BDEF relationship can depend on whether local heterogeneity or regional species pool differences underlie diversity differences, with positive BDEF relationships only expected in the latter case [26]. In no case did we find a significant positive relationship between local resource heterogeneity and species diversity, suggesting that heterogeneity did not underlie the effects of diversity in the three systems examined. This allowed a valid examination of how broader scale (site-to-site) differences in diversity interact with smaller scale variation in resource abundance (within-site heterogeneity) to determine functional processes in each focal system. Our finding that the slope of the BDEF relationship increased with resource heterogeneity could also not be attributed to the potentially confounding, yet commonly observed, influence of resource abundance on function (e.g., [25,31]), because the standardized total effect (the sum of direct and indirect effects, while controlling for all other variables in the model, standardized for differences in the magnitude of different units) of all of the resource abundance measures on function was lower than the diversity × heterogeneity interaction in the SEMs from all three systems (Tables S7, S11, and S13). Finally, it may be argued that in real world ecosystems, species composition will co-vary with diversity, potentially confounding BDEF relationships. We tested changes in community composition (Text S3), and found that this was not confounding the effects of diversity in our system (Figure 3, Text S4). Nevertheless, nonrandom extinctions and altered community composition may be an additional mechanism through which biodiversity loss affects ecosystem functioning [7,40], particularly if sampling effects are also important in real systems [17]. This was supported by the composition-mediated effects of diversity in the parasitoid system, and species composition additionally contributed to differences in belowground biomass for the plant communities (Figure 3A, Tables S6 and S7).

Thus, after accounting for likely confounding factors, the data still strongly support a positive diversity-function relationship, which increases when resources are heterogeneously distributed. Distinguishing between the mechanisms underlying positive diversity effects (e.g., selection, facilitation, or complementarity) is difficult in this kind of field study, and requires future research to experimentally manipulate diversity and heterogeneity. However, given the strong influence of resource heterogeneity in mediating the BDEF relationship, complementarity seems to be a likely candidate, meriting discussion in the context of each of the three systems examined.

In the temperate grassland system, the effects of plant diversity on belowground standing biomass became most evident in ecosystems where essential nutrients (particularly NO_3_^−^, P, and C) were heterogeneously distributed, which is the norm in most ecosystems [47], and allows complementary exploitation of the resulting niche spaces [45]. The contrasting patterns between above- and belowground biomass may be explained by root foraging patterns that make belowground biomass particularly susceptible to nutrient heterogeneity [47]. In contrast, aboveground biomass may respond more to absolute nutrient concentrations [25,47] than to heterogeneity, and this was supported by the best fitting SEM for aboveground biomass (Tables S8 and S9), where each of the soil nutrient availability factors had greater total and standardized total effects on biomass than did soil heterogeneity (or the diversity × heterogeneity interaction, which had no effect). Therefore, our finding that belowground biomass responded more strongly to diversity under high heterogeneity in NO_3_^−^ is congruent with previous experiments.

Parasitoids have long been known to respond strongly to heterogeneity in the availability of underlying host resources (e.g., [48,49]), and here too, responses are often highly species specific. For example, different parasitoid species attacking a shared host can differ in their density dependence, with some species parasitizing a greater proportion of hosts at low densities and others showing the opposite pattern [39,50]. These different behavioral responses of parasitoid species to host density likely reflect differences in species characteristics such as dispersal ability, degree of specialization and search behavior [39,50]. In the host-parasitoid system of coastal Ecuador, we found that the positive effect of diversity on parasitism rates increased with increasing within-plot spatial heterogeneity in host abundance. Again this is consistent with the idea that different species were more effective at exploiting subpopulations at different densities resulting in complementary resource use in heterogeneous systems.

Different pollinator groups have been shown to be attracted by different floral designs, forms, abundances, and densities, and this can depend largely on their foraging behavior [38,51]. For example, Thompson found that butterflies and hover flies responded strongly to the number of open flowers in a patch (i.e., resource availability) [38], whereas other pollinators such as short-tongued bees responded more strongly to other floral characteristics. Similarly, mass recruiting species such as honeybees (*Apis*) are known to respond quickly to changes in floral resource availability through communication between workers, and tend to be attracted to patches of high resource abundance or density [52,53], while bumblebee (Bombus spp.) workers forage more independently and may be more likely to visit rarer or more sparsely distributed resources. Although coffee plants attract a diversity of solitary species, social bees such as honeybees and stingless bees are almost absent when flowering is scarce [34]. Displacement by aggressive social bees of inferior competitors from large floral resource patches may also cause rarer solitary bees to utilize smaller patches that are less attractive to their competitors [54]. Given that different pollinator species can vary in their foraging behavior [55] and responses to patterns in the floral community, increasing pollinator diversity would be expected to result in more complete utilization of host plant patches and thus greater function in heterogeneous systems. This is consistent with results from our coffee agroforest system, in which the slope of the relationship between bee diversity and rates of coffee pollination increased with increasing spatial heterogeneity in the abundance of coffee flowers.

### Conclusions

Alternative approaches to studying the relationships between biodiversity and processes or functions have often led to contrasting results, with a positive relationship generally found in smaller scale manipulative experiments and more variable or negative relationships found in larger scale observational studies [56–58]. Thus, while experimentalists argue that effects of biodiversity can only be understood via direct manipulations of diversity (indeed such experiments are obviously critical for testing certain causal mechanisms, such as facilitation or complementarity), others have begun to question the relevance of such experiments for assessing the importance of biodiversity in real world systems [40,56]. Ultimately, such polarization is inimical to ecological progress, and full understanding of real world systems and their underlying mechanisms requires a suite of theoretical, empirical, and observational techniques [59].

Our results are congruent with predictions from theory, previous experiments, and recent field studies, showing that biodiversity can have strong effects on ecosystem functioning even in real, nonexperimental habitats. Our study presents statistical correlations and can therefore not be used to infer causation or effects of biodiversity loss per se (differences in diversity among our sites may reflect factors other than species losses). Nevertheless, our results provide strong empirical evidence from different ecosystems that the BDEF relationship can depend on the habitat context, requiring resource heterogeneity for a positive BDEF relationship to occur. Previous controlled experimental studies using random species assemblages without consideration of environmental heterogeneity, may not have contained the varied niche space required for full expression of diversity effects [36]. Therefore the positive relationships observed in experiments may have even been underestimates of the strength of the BDEF relationship, and its importance to real world ecosystems, and future experiments that explicitly consider heterogeneity are needed.

Recent years have seen enormous strides in understanding of the BDEF relationship. Our results provide generality, by elucidating the real world conditions under which previously established patterns are likely to occur. Combined with calls from theoretical work, our results suggest that expanding research to include more realistic conditions, such as heterogeneous habitats or resources, will be critical towards further understanding the BDEF relationship. Only a synergy between theoretical, experimental, and observational approaches will be able to untangle the real world importance of diversity for ecosystem functioning, and allow us to fully understand the perils of biodiversity loss.

## Materials and Methods

### Plant biomass: Study sites and sampling.

The study was conducted in 19 semi-natural grasslands in the Thüringer Schiefergebirge/Frankenwald, in central Germany [41]. In each grassland, four soil samples were collected in mid May 2002 to characterize soil spatial heterogeneity. Part of the sample (∼10 g) was extracted with 50 ml 1 M KCl for 60 min on the day of sampling (May 15, 2002). KCl extracts were filtered and frozen at −20 °C and later analyzed using a continuous flow analyzer (SAN Plus, Skalar) for NH_4_^+^ and NO_3_^−^, and an ICP-AES (Optima 3300 DV, Perkin-Elmer) for Ca^2+^. The remaining soil was dried at 35 °C and extracted for 1 h using a 1 M calcium acetate lactate (CAL) solution. CAL extracts were analyzed with ICP-AES for P, K^+^, Mg^2+^, and SO_4_^2−^. Soil pH was measured in a water extract. To measure soil C:N ratio, total N and total C concentrations, dry soil was ground and analyzed with an elemental analyzer (Vario EL II, Elementar).

A 3 × 3-m area was sampled in each grassland for plant biodiversity [41]. We followed Kahmen et al. [41] by calculating plant diversity (exponential Shannon-Wiener) as H′ = −Σ(*p_i_*)(ln *p_i_*), where *p_i_* = species cover/sum of cover for all species. Aboveground standing biomass was determined in eight 25 × 50-cm plots per grassland. Samples were collected in late June and early September 2003, following the local management regime. Biomass was clipped 2 cm above the ground. Belowground standing biomass was determined using soil cores (4.3 cm diameter, 10 cm length). Four cores were collected in the central sampling area of each grassland, at the end of the growing season (mid September). The roots were removed from the soil, dried, and weighed.

Soil heterogeneity was characterized by the within-plot CV (the standard deviation as a proportion of the mean) of each of the soil variables (NH_4_^+^, NO_3_^−^, Ca^2+^, P, K^+^, Mg^2+^, Na, C:N ratio, total N, N_min_, total C, and pH_(H2O)_). As there were a large number of intercorrelated soil variables relative to our number of replicates, and to alleviate the need for subjective decisions about which soil characteristics may be important, we used a PCA in Statistica 6.1 (Statsoft) to reduce these 12 variables to five factors with an eigenvalue greater than 1, which cumulatively explained over 80% of the variation in soil heterogeneity (Tables S1 and S2). PCA is a technique for simplifying a multidimensional dataset by reducing it to fewer orthogonal dimensions for analysis, while retaining those characteristics that contribute most to its variance. This same technique was used to reduce the absolute values of each soil variable (i.e., not the CV) to four orthogonal factors (Tables S4 and S5) for structural equation modeling.

### Parasitism: Study sites and sampling.

The 48 study plots were spread across three cantons in the region of Jipijapa (17 N 546,800 m, E 984,9274 m altitude 259 m), within the province of Manabi, Southwest Ecuador (for individual plot location details and a full description of the region see [60]). This area falls within the Choco-Manabi biodiversity hotspot, but large-scale agricultural conversion threatens the local biodiversity and the ecosystem services it provides [11]. There were 12 rice, 12 pasture, 12 coffee, six abandoned coffee, and six forest plots.

Nine standardized trap nests were positioned (in a 3 × 3 grid, 25 m between adjacent traps) in the center of each of the 48 plots, to provide nesting sites for naturally occurring communities of bees and wasps (hosts) and their natural enemies [11,60]. A PVC tube with a length of 22 cm and a diameter of 15 cm formed the outer case of the nest. Internodes of reeds Arundo donax L. (Poaceae) with varying diameter (2–20 mm) and a length of 20 cm were inserted into this tube and provided the nesting sites for bees and wasps. Exposure of standardized trap nests is similar to the exposure of other resources, e.g., phytometer plants, but because the guild of aboveground cavity-nesting species reproduces in these traps, the problem of species appearing as “tourists” in samples is eliminated. The traps were evaluated every month from June 2003 to October 2004. Occupied reeds were opened and the host larvae were reared to maturity for positive identification and to detect the presence of any parasitoids. Parasitism rate was defined as the proportion of host larvae attacked by parasitoids. Data from each of the nine traps per plot were pooled across all months for analyses.

Of all the potential host species found in our traps [60], the one with the greatest diversity of parasitoids attacking it (six species) was Pseudodynerus sp. (Hymenoptera: Eumenidae). This was the only host species found in every plot (*n* = 48), it was the second most abundant of all the host species (6,884 individuals, 487 of which were parasitized), and had the most even distribution among habitat types—allowing statistical analyses in all habitats. We therefore used this species as our focal host for this study. The natural enemies attacking *Pseudodynerus* were gregarious ectoparasitoids, solitary ectoparasitoids, endoparasitoids, or kleptoparasites [11], but here we refer to them collectively as “parasitoids.”

To quantify host heterogeneity (patchiness) in each plot, we used the CV in host (*Pseudodynerus*) abundance between the nine trap nests within one plot. Abundance was measured as the number of *Pseudodynerus* larvae in all occupied reeds over the entire sampling period.

### Pollination: Study sites and sampling.

The 24 study plots were spread across the agricultural landscape of the villages Wuasa, Watumaeta, Alitupu, and Kaduwaa at the margin of the Lore-Lindu-National Park, Central Sulawesi (Indonesia) (for site details see [15]). Sulawesi is a biodiversity hotspot, including many endemic species in and around the Lore-Lindu-National Park, but ongoing agricultural conversion at the forest margins threatens the endemic species and the local diversity.

We randomly selected four coffee (Coffea arabica L.) plants in each of the 24 coffee agroforests, and used one branch per plant for an open pollination treatment, and another one for a bagged pollination treatment. Bags of nylon mesh gauze (10 μm) were fixed on the bagged treatment coffee branches 1–6 d before blooming, to exclude pollen transferred by insects or wind. Tanglefoot was applied on the branch beneath the bagged flowers to exclude ants. We counted and tagged flowers on the observed bagged and open branches. After flowering, we removed the bags, and 5 wk later developing fruits were counted (see also [15]). As fruit set can vary significantly with plant quality and local soil and microclimatic conditions, we defined pollination benefit as the proportion of flowers that set fruit from the open pollination treatment, minus the proportion that set fruit in the bagged control treatment. This controlled for between-plant variability in fruit set due to genetic or environmental factors.

Because of time constraints during the short flowering period, flower-visiting bees and resulting fruit were sampled on only three of the four coffee shrubs in each site, following the methodology of Klein et al. [15]. We counted the flowering branches on each of these three shrubs, and the between-shrub CV in these values was used as an estimate of resource heterogeneity. We collected flower-visiting bees for 25 min on each of the three coffee plants, and further estimates of plot-scale bee diversity were obtained by sweep-netting for 5 min.

### Statistical analyses.

Unless stated otherwise, analyses were conducted in R v. 2.3.1 (R Development Core Team, http://www.r-project.org). Model residuals were tested for adherence to a normal distribution and homogeneity of variances, and response variables were transformed when necessary.

We tested the effects of plant diversity and soil heterogeneity on above and belowground standing biomass using multiple regressions. The initial (maximal) model contained standing biomass as the response variable, and plant diversity and all five soil heterogeneity factors from the PCA were included as continuous predictors. Interaction effects between plant diversity and each of the soil heterogeneity factors were also included in the model. Separate models were used for above and belowground biomass. Models were simplified by removing nonsignificant interaction terms then main effects, until model fit (assessed using Akaike Information Criterion [AIC]), no longer improved. When model fit did not differ significantly between two competing models (the difference in AIC score was <2), we selected the most parsimonious model (the model with fewest parameters). Full details of maximal and minimal adequate above- and belowground models are provided in Text S1A and S1B, respectively.

The proportion of *Pseudodynerus* larvae parasitized per plot was arcsine square root transformed prior to analyses to meet assumptions of the parametric tests. We tested the effects of habitat type, natural enemy diversity, and host heterogeneity on rates of parasitism using ANCOVA, with Type I sums of squares. No model simplification was needed, as we only included one measure of heterogeneity, compared with five factors in the plant model above. Habitat type entered the model first, then parasitoid species richness, then host heterogeneity (within-plot CV in abundance), followed by the main interaction of interest (host heterogeneity × parasitoid species richness interaction effect), and a three-way interaction between host heterogeneity, parasitoid richness, and habitat type (to determine if the mediating effect of host heterogeneity varied across habitats). To be conservative, we excluded all zero values from analyses, as incidents of zero diversity and/or zero parasitism could drive a positive diversity function relationship by default, and their inclusion only made the effects presented in the results and graphs even stronger.

We tested the effects of bee diversity and flower heterogeneity on pollination success (percent fruit set of open-bagged control flowers) using a multiple regression with Type I sums of squares. Bee richness entered the model first, followed by flower heterogeneity and the interaction of these two variables.

In addition to the above analyses, we applied NMDS ordination techniques to test for correlations between NMDS axes (community composition of plants/parasitoids/pollinators) and diversity in each of our systems. These analyses (Text S3) showed that species composition did not confound the effects of species diversity.

Although the ANCOVA and multiple regressions above tested our hypothesized effects, it was also possible that diversity responded to another variable that also has an effect on function. Similarly, resource abundance may have had complex indirect effects, possibly mediated through diversity, which was also correlated with species composition in the parasitoid and pollinator communities. Therefore, to control for these possible confounding variables, we used SEMs, performed in Amos v.16.0.1 (Amos Development Corporation, http://amosdevelopment.com). We included the above NMDS axes along with resource abundance, heterogeneity, and diversity variables in the SEMs. For each system we constructed an initial model with a variety of pathways allowing resource abundance and heterogeneity to affect diversity, composition, and function. We then simplified these models down to a final model using AIC scores (Text S4), and present the final simplified models in Figure 3.

## Supporting Information

Figure S1 Initial Belowground Plant Model Original SEM before model simplification. Also number of parameters, AIC and Browne-Cudeck criterion (BCC) scores, and minimum discrepancy (Cmin) for initial and final simplified models.
(55 KB DOC)

Figure S2 Initial Aboveground Plant Model Original SEM before model simplification. Also number of parameters, AIC and BCC scores, and Cmin for initial and final simplified models.
(53 KB DOC)

Figure S3 Initial Parasitoid Model Original SEM before model simplification. Also number of parameters, AIC and BCC scores, and Cmin for initial and final simplified models.
(51 KB DOC)

Figure S4 Initial Pollinator Model Original SEM before model simplification. Also number of parameters, AIC and BCC scores, and Cmin for initial and final simplified models.
(53 KB DOC)

Table S1 PCA Statistics Twelve factors that explained all of the variance in heterogeneity of the original 12 soil parameters.
(59 KB DOC)

Table S2 PCA Factor Loadings Correlation between the five PCA factors used in analyses and the original soil heterogeneity variables.
(66 KB DOC)

Table S3 The Correlation Coefficient (*r*) and Significance Level (*p*) for Pearson Correlations between Soil Parameters and Significant Soil Heterogeneity Factor 5 (46 KB DOC)

Table S4 PCA Statistics Twelve factors that explained all of the variance in the absolute values of the 12 soil parameters (i.e., not variability as in Tables S1 and S2).
(59 KB DOC)

Table S5 PCA Factor Loadings Correlation between the four PCA factors used in SEM analyses and the original soil variables.
(58 KB DOC)

Table S6 Total Effects for Final Belowground Plant Model Unstandardized total effects for final belowground plant SEM.
(48 KB DOC)

Table S7 Standardized Total Effects for Final Belowground Plant Model Standardized total effects for final belowground plant SEM.
(48 KB DOC)

Table S8 Total Effects for Final Aboveground Plant Model Unstandardized total effects for final aboveground plant SEM.
(51 KB DOC)

Table S9 Standardized Total Effects for Final Aboveground Plant Model Standardized total effects for final aboveground plant SEM.
(50 KB DOC)

Table S10 Total Effects for Final Parasitoid Model Unstandardized total effects for final parasitoid SEM.
(52 KB DOC)

Table S11 Standardized Total Effects for Final Parasitoid Model Standardized total effects for final parasitoid SEM.
(51 KB DOC)

Table S12 Total Effects for Final Pollinator Model Unstandardized total effects for final pollinator SEM.
(47 KB DOC)

Table S13 Standardized Total Effects for Final Pollinator Model Standardized total effects for final pollinator SEM.
(47 KB DOC)

Text S1 Summary of Models Examining the Effects of Diversity and Heterogeneity on Function Maximal and minimum adequate models for plant (A) belowground and (B) aboveground biomass. ANOVA tables for (C) parasitoid and (D) pollinator models.
(55 KB DOC)

Text S2 Soil Heterogeneity and Nutrient Availability (44 KB DOC)

Text S3 Community Composition Analyses Details of NMDS analyses and correlations between NMDS axes and diversity in each of our systems.
(61 KB DOC)

Text S4 SEM Details of SEM analysis.
(45 KB DOC)

## Abbreviations

AIC: Akaike Information Criterion
ANCOVA: Analysis of Covariance
BCC: Browne-Cudeck criterion score
BDEF: biodiversity-ecosystem functioning
Cmin: minimum discrepancy
CV: coefficient of variation
NMDS: nonmetric multidimensional scaling
PCA: principle component analysis
SEM: structural equation model
