# Supplementary material for: Resource Heterogeneity Moderates the Biodiversity-Function Relationship in Real World Ecosystems
Source: PLoS Biol. 2008 May 20;6(5):e122. doi: 10.1371/journal.pbio.0060122 (PMC2386840; doi:10.1371/journal.pbio.0060122)
Supplement: Supplementary file 1 [file pbio.0060122.sd001.doc]

**Text S1:**

**1A) Belowground biomass:**

Maximal model (containing plant diversity, five soil heterogeneity factors from PCA, and interactions between plant diversity and heterogeneity factors as predictors).

```
Coefficients:
                         Estimate Std. Error t value Pr(>|t|)
(Intercept)             1.986e+02  3.667e+02   0.542   0.6049
plant.divers            6.027e+01  3.240e+01   1.860   0.1052
soil.het1              -6.056e+02  4.522e+02  -1.339   0.2223
soil.het2               6.676e+02  3.299e+02   2.024   0.0827
soil.het3              -2.591e+01  3.562e+02  -0.073   0.9440
soil.het4              -3.169e+01  2.094e+02  -0.151   0.8840
soil.het5              -1.139e+03  6.583e+02  -1.731   0.1271
plant.divers:soil.het1  3.116e+01  2.278e+01   1.368   0.2137
plant.divers:soil.het2 -5.152e+01  2.407e+01  -2.141   0.0696
plant.divers:soil.het3 -3.795e-02  2.851e+01  -0.001   0.9990
plant.divers:soil.het4  1.267e+01  1.529e+01   0.829   0.4346
plant.divers:soil.het5  9.770e+01  5.547e+01   1.761   0.1216
---
Signif. codes:  0 '***' 0.001 '**' 0.01 '*' 0.05

Residual standard error: 200.9 on 7 degrees of freedom
Multiple R-Squared: 0.745,      Adjusted R-squared: 0.3444
F-statistic:  1.86 on 11 and 7 DF,  p-value: 0.2104
```

Minimal adequate model:

```
Coefficients:
                       Estimate Std. Error t value Pr(>|t|)
(Intercept)              125.15     226.69   0.552  0.58902
plant.divers              54.05      18.04   2.996  0.00904 **
soil.het5               -616.34     235.27  -2.620  0.01933 *
plant.divers:soil.het5    60.69      20.81   2.916  0.01064 *
---
Signif. codes:  0 '***' 0.001 '**' 0.01 '*' 0.05 '.' 0.1 ' ' 1

Residual standard error: 207.8 on 15 degrees of freedom
Multiple R-Squared: 0.4156,     Adjusted R-squared: 0.2987
F-statistic: 3.555 on 3 and 15 DF,  p-value: 0.04009
```

**1B) Aboveground biomass:**

Maximal model (containing plant diversity, five soil heterogeneity factors from PCA, and interactions between plant diversity and heterogeneity factors as predictors).

```
Coefficients:
                       Estimate Std. Error t value Pr(>|t|)
(Intercept)            535.3830   336.6722   1.590    0.156
plant.divers            -4.7591    29.7479  -0.160    0.877
soil.het1              170.0041   415.1191   0.410    0.694
soil.het2              196.9513   302.8702   0.650    0.536
soil.het3              482.6106   326.9768   1.476    0.183
soil.het4               31.9617   192.2193   0.166    0.873
soil.het5              395.7568   604.3784   0.655    0.534
plant.divers:soil.het1  -3.8456    20.9169  -0.184    0.859
plant.divers:soil.het2 -10.0107    22.0969  -0.453    0.664
plant.divers:soil.het3 -38.4684    26.1745  -1.470    0.185
plant.divers:soil.het4  -0.9989    14.0416  -0.071    0.945
plant.divers:soil.het5 -25.6033    50.9290  -0.503    0.631

Residual standard error: 184.5 on 7 degrees of freedom
Multiple R-Squared: 0.4251,     Adjusted R-squared: -0.4782
F-statistic: 0.4706 on 11 and 7 DF,  p-value: 0.873
```

Minimal adequate model: (Lowest AIC score, but remained non-significant)

```
Coefficients:
             Estimate Std. Error t value Pr(>|t|)
(Intercept)   631.572    123.840   5.100  8.9e-05 ***
plant.effdiv   -8.933      8.749  -1.021    0.322
---
Signif. codes:  0 '***' 0.001 '**' 0.01 '*' 0.05 '.' 0.1 ' ' 1

Residual standard error: 151.5 on 17 degrees of freedom
Multiple R-Squared: 0.05778,    Adjusted R-squared: 0.00236
F-statistic: 1.043 on 1 and 17 DF,  p-value: 0.3215
```

**1C) Parasitoid model:**

```
Analysis of Variance Table

Response: proportion parasitised
                               Df   Sum Sq  Mean Sq F value    Pr(>F)
Habitat type                    4 0.055273 0.013818  2.4107 0.0719 .
Parasitoid diversity            1 0.087968 0.087968 15.3470 0.0005***
Host heterogeneity              1 0.008009 0.008009  1.3973 0.2468
Diversity*heterogeneity         1 0.029809 0.029809  5.2004 0.0301*
Habitat*diversity*heterogeneity 4 0.022755 0.005689  0.9925 0.4273
Residuals                      29 0.166227 0.005732
---
Signif. codes:  0 '***' 0.001 '**' 0.01 '*' 0.05 '.' 0.1 ' ' 1
```

**1D) Pollinator model:**

```
Analysis of Variance Table

Response: Fruit set change
                    	Df  Sum Sq Mean Sq F value   Pr(>F)
Pollinator diversity    1  666.66  666.66  8.9482 0.007215 **
Flower heterogeneity    1   75.94   75.94  1.0193 0.324737
Diversity*heterogeneity 1  701.58  701.58  9.4170 0.006059 **
Residuals              20 1490.04   74.50
---
```

Signif. codes: 0 '***' 0.001 '**' 0.01 '*' 0.05 '.' 0.1 ' ' 1
