# Supplementary material for: Resource Heterogeneity Moderates the Biodiversity-Function Relationship in Real World Ecosystems
Source: PLoS Biol. 2008 May 20;6(5):e122. doi: 10.1371/journal.pbio.0060122 (PMC2386840; doi:10.1371/journal.pbio.0060122)
Supplement: Supplementary file 2 [file pbio.0060122.sd002.doc]

**Text S2: Soil heterogeneity and nutrient availability**

Previous studies (see main text for citations) have shown that nutrient availability can affect the diversity-productivity relationship. This may lead to the reasonable contention that the relationships with soil heterogeneity we present are in fact due to positive or negative correlations between soil heterogeneity and the availability of certain nutrients. To test this possibility, we used a Pearson’s correlation analysis to test for correlations between the significant soil heterogeneity PCA factor 5 and each of the 12 soil variables (raw values/concentrations, not heterogeneity). We found no significant correlations at a Bonferroni corrected alpha of 0.00416. Although Bonferroni corrections have been criticised (Moran 2003), the probability of two variables out of 12 having *p* values below 0.047 (see C and P in Table S3) can be calculated using a Bernoulli process (Moran 2003, see also main text), and in our case this probability was 0.09 – still higher than the alpha of 0.05 conventionally used in scientific studies.

Nevertheless, resource abundance in general may be a confounding factor in any field study of the BDEF relationship, therefore it was included as an exogenous variable in the SEM analyses below (Text S4). Resource abundance was easily measured for the parasitoid-host system in Ecuador (abundance of host larvae) and the pollinator-plant system in Indonesia (abundance of coffee flowers). However, in the German grasslands, a variety of nutrients are present, and the ‘abundance’ (concentration) of different nutrients may be intercorrelated. Therefore, to take into account this intercorrelation and reduce the number of variables for analysis, we conducted another PCA as above, but using the concentrations of each soil variable, rather than their within-site variability. This allowed us to reduce the 12 soil variables to 4 orthogonal factors that cumulatively explained over 81% of the variance in the soil variables (Tables S4 and S5). These factors were used in the SEM analyses below (Text S4).

**Reference:**

Moran, M.D. 2003. Arguments for rejecting the sequential Bonferroni in ecological studies. Oikos 100, 403-405.
