# Supplementary material for: Resource Heterogeneity Moderates the Biodiversity-Function Relationship in Real World Ecosystems
Source: PLoS Biol. 2008 May 20;6(5):e122. doi: 10.1371/journal.pbio.0060122 (PMC2386840; doi:10.1371/journal.pbio.0060122)
Supplement: Supplementary file 3 [file pbio.0060122.sd003.doc]

**Text S3: Community composition analyses**

In real-world ecosystems, extinctions of species are non-random. This was not a problem for BDEF experiments, as diversity treatments were created using random assemblages of species. In real-world studies, however, habitats that differ in their diversity, may also differ in the species that comprise that diversity, and this change in composition must be controlled for in any assessment of diversity effects on ecosystem functioning.

To analyse compositional differences among plants/parasitoids/pollinators, we applied non-metric multidimensional scaling (NMDS) ordination techniques using the program PC-ORD version 4.25 (McCune & Mefford 1997). NMDS is an iterative search for ranking and placement of n entities (samples) in k dimensions (ordination axes) that minimizes the stress of the k-dimensional configuration. The ‘‘stress’’ value is a measure of departure from monotonicity in the relationship between the dissimilarity (distance) in the original p-dimensional space and in the reduced k-dimensional ordination space (Clarke 1993). NMDS is therefore used to find a configuration in a given number of dimensions, which preserves rank-order dissimilarities in species composition as closely as possible, such that distance along a NMDS axis corresponds to relative difference in community composition. As distance measure, the Bray-Curtis coefficient was used (also known as Sørensen or Czekanowski coefficient), which is one of the most robust measures for this purpose (Faith et al. 1987).

We then tested for correlations between NMDS axes and diversity in each of our systems, to determine whether species composition correlates with diversity in our sites:

**Plants (Germany)**

Number of axes: 2

Number of iterations: 43

Stress for 2-dimensional solution: axis 1= 20.08, axis 2= 12.45 (final stress value: 12.45)

|  | NMDS axis 1 | NMDS axis 2 | Plant diversity |
| --- | --- | --- | --- |
| NMDS axis 1 | 1 |  |  |
| NMDS axis 2 | **0.528*** | 1 |  |
| Plant diversity | -0.349 | -0.301 | 1 |

Correlations from Pearson, N = 19. * = significant at 0.05, ** = significant at 0.01

**Parasitoids (Ecuador)**

Number of axes: 2

Number of iterations: 400

Stress for 2-dimensional solution: axis 1= 27.91 , axis 2= 12.58 (final stress value: 12.58)

|  | NMDS axis 1 | NMDS axis 2 | Parasitoid richness |
| --- | --- | --- | --- |
| NMDS axis 1 | 1 |  |  |
| NMDS axis 2 | -0.035 | 1 |  |
| Parasitoid richness | **0.644**** | 0.036 | 1 |

Correlations from Pearson, N = 41. * = significant at 0.05, ** = significant at 0.01

**Pollinators (Indonesia)**

Number of axes: 3

Number of iterations: 196

Stress for 2-dimensional solution: axis 1= 26.49, axis 2= 13.52, axis 3= 7.92 (final stress value: 8.14)

|  | NMDS axis 1 | NMDS axis 2 | NMDS axis 3 | Pollinator richness |
| --- | --- | --- | --- | --- |
| NMDS axis 1 | 1 |  |  |  |
| NMDS axis 2 | 0.099 | 1 |  |  |
| NMDS axis 3 | 0.002 | 0.215 | 1 |  |
| Pollinator richness | 0.256 | -0.183 | **-0.615**** | 1 |

Correlations from Pearson, N = 24. * = significant at 0.05, ** = significant at 0.01

Diversity did not correlate with composition in the plant communities, however, parasitoid diversity was significantly correlated with NMDS axis 1, and pollinator diversity was significantly correlated with NMDS axis 3.

**References:**

Clarke, K.P., 1993. Non-parametric multivariate analyses of changes in community structure. Aust. J. Ecol. 18, 117– 143.

Faith, D. P., Minchin, P. R. and Belbin, L. 1987. Compositional dissimilarity as a robust measure of ecological distance. Vegetatio 69, 7–68.

McCune, B., Mefford, M.J., 1997. PC-ORD. Multivariate Analysis of Ecological Data, Version 3.0. MjM Software Design, Gleneden Beach.
