# Supplementary material for: Resource Heterogeneity Moderates the Biodiversity-Function Relationship in Real World Ecosystems
Source: PLoS Biol. 2008 May 20;6(5):e122. doi: 10.1371/journal.pbio.0060122 (PMC2386840; doi:10.1371/journal.pbio.0060122)
Supplement: Supplementary file 4 [file pbio.0060122.sd004.doc]

**Text S4 Structural Equation Modelling (SEM)**

In a field study such as ours, diversity may be a predictor of function, but it may also respond to another variable that also has an effect on function. Furthermore, resource abundance may have complex indirect effects, possibly mediated through diversity, which was also correlated with species composition in the parasitoid and pollinator communities (quantified using the NMDS axes in Text S3). Therefore, the apparent effect of diversity could in fact be due to shifts in species composition, and the effects of diversity and the diversity*heterogeneity interaction may not be significant after controlling for all of these different confounding variables.

To distinguish between these potential causal pathways and control for these possible confounding variables, we used SEM, performed in Amos v.16.0.1 (Amos Development Corporation http://amosdevelopment.com). For each system we constructed an initial model (presented below) with a variety of pathways allowing resource abundance and heterogeneity to affect diversity. We also included pathways from diversity to species composition to function, allowing for the effect of diversity to be mediated via shifts in composition, rather than diversity per se. The interaction term was derived by cross-multiplying “centred” (deviation) scores of the main effects (Kline & Dunn 2000). We allowed for species composition, resource abundance, and habitat type (parasitoid model only) to affect this interaction term of interest. All paths in the original model were treated as optional, and were thus able to be removed during model simplification. We used the specification search function, to test all subsets of our initial model using maximum likelihood estimation. As a final model we selected the model with a subset of the parameters in the initial model that included at least one predictor of function (plant biomass, parasitism or pollination respectively), and had the lowest AIC (Akaike Information Criterion) score. When multiple models did not differ significantly in AIC (less than 2 units difference), we selected the most parsimonious model (fewest parameters). Below we present the diagram for the initial model, and present the final most parsimonious model in main text Fig. 3. Values adjacent to paths indicate unstandardized direct effects, with significance indicated by * = P< 0.05, ** = P<0.01, ***=P<0.001. For each model we present the number of distinct parameters being estimated, Cmin (the minimum value of the discrepancy *C*), AIC score and BCC (the Browne-Cudeck Criterion, which was developed specifically for analysis of moment structures and gives a slightly greater penalty for model complexity than AIC). Unstandardized (absolute) and standardized (accounting for different unit sizes in different predictors) total effects (direct and indirect combined) are presented in tables below each final model. In the total effects tables, the column variable is explaining the row variable.

**Reference:**

Kline T.J.B. and Dunn, B. (2000) Analysis of interaction terms in structural equation models: a non-technical demonstration using the deviation score approach. Can. J. Behav. Sci. 32, 127-132.
