# Supplementary material for: Resource Heterogeneity Moderates the Biodiversity-Function Relationship in Real World Ecosystems
Source: PLoS Biol. 2008 May 20;6(5):e122. doi: 10.1371/journal.pbio.0060122 (PMC2386840; doi:10.1371/journal.pbio.0060122)
Supplement: Supplementary file 5 [file pbio.0060122.sg001.doc]

**Figure S1: Initial belowground plant model**

The four Soil factors are combined measures of 12 soil parameters obtained by a PCA (see Table S4 above). “Soil Het.” is the soil heterogeneity factor 5 (from PCA on within-plot CV in soil parameters), which was found to be significant in the general linear model for belowground biomass (see main text). The “Div*Het Interaction” is the interaction of interest between diversity and soil heterogeneity. Plant composition 1 and 2 are the NMDS axes from the species composition ordinations in Supplementary Results 3 above.

| **Parameters** | **Cmin** | **AIC** | **BCC** |
| --- | --- | --- | --- |
| 45 | 37.136 | 127.136 | 268.565 |

**Final belowground plant model (Fig. 3 A)**

| **Parameters** | **Cmin** | **AIC** | **BCC** |
| --- | --- | --- | --- |
| 34 | 43.869 | 111.869 | 218.726 |

Soil

Factor 1

Soil

Factor 2

Soil

Factor 3

Soil

Factor 4

Soil

Het.

Belowground Biomass

Plant Diversity

Plant Composition 1

Plant Composition 2

Div* Het Interaction

e2

e6

e5

e1

e3

e4
