# Supplementary material for: Resource Heterogeneity Moderates the Biodiversity-Function Relationship in Real World Ecosystems
Source: PLoS Biol. 2008 May 20;6(5):e122. doi: 10.1371/journal.pbio.0060122 (PMC2386840; doi:10.1371/journal.pbio.0060122)
Supplement: Supplementary file 6 [file pbio.0060122.sg002.doc]

**Figure S2: Initial aboveground plant model**

| **Parameters** | **Cmin** | **AIC** | **BCC** |
| --- | --- | --- | --- |
| 45 | 37.140 | 127.140 | 268.568 |

**Final aboveground plant model (Fig. 3 B)**

| **Parameters** | **Cmin** | **AIC** | **BCC** |
| --- | --- | --- | --- |
| 29 | 52.647 | 110.647 | 201.790 |

Note: model was unstable

**Initial Model:**

Soil

Factor 1

Soil

Factor 2

Soil

Factor 3

Soil

Factor 4

Soil

Het.

Aboveground Biomass

Plant Diversity

Plant Composition 1

Plant Composition 2

Div* Het Interaction

e2

e6

e5

e1

e3

e4
