# Supplementary material for: Resource Heterogeneity Moderates the Biodiversity-Function Relationship in Real World Ecosystems
Source: PLoS Biol. 2008 May 20;6(5):e122. doi: 10.1371/journal.pbio.0060122 (PMC2386840; doi:10.1371/journal.pbio.0060122)
Supplement: Supplementary file 7 [file pbio.0060122.sg003.doc]

**Figure S3: Initial parasitoid model**

“Host Het.” is the heterogeneity in host abundance (within-plot CV in abundance). The “Div*Het Interaction” is the interaction of interest between parasitoid diversity and heterogeneity in host abundance. Plant composition 1 and 2 are the NMDS axes from the species composition ordinations in Supplementary Results 3 above. The proportion parasitized is arcsin square root transformed, as in the GLM in the main text.

Minimisation was unsuccessful, reliable parameter estimates could not be obtained.

**Final parasitoid model (Fig. 3 C)**

| **Parameters** | **Cmin** | **AIC** | **BCC** |
| --- | --- | --- | --- |
| 24 | 48.707 | 96.707 | 108.076 |

**Initial Model:**

Host Abundance

Host

Het.

Proportion parasitised

Parasitoid Diversity

Parasitoid Composition 1

Parasitoid Composition 2

Div* Het Interaction

e3

e2

e1

e5

e6

e4

Habitat Type

e7
