# Supplementary material for: Resource Heterogeneity Moderates the Biodiversity-Function Relationship in Real World Ecosystems
Source: PLoS Biol. 2008 May 20;6(5):e122. doi: 10.1371/journal.pbio.0060122 (PMC2386840; doi:10.1371/journal.pbio.0060122)
Supplement: Supplementary file 8 [file pbio.0060122.sg004.doc]

**Figure S4: Initial pollinator model**

“Flower Het.” is the heterogeneity in abundance of coffee flowers (between-shrub CV in abundance). The “Div*Het Interaction” is the interaction of interest between pollinator diversity and heterogeneity in flower abundance. Pollinator composition 1-3 are the NMDS axes from the species composition ordinations in Supplementary Results 3 above. “Pollination benefit” was quantified as the proportion of flowers that set fruit from the open pollination treatment, minus the proportion that set fruit in the bagged control treatment (as in the GLM in the main text).

| **Parameters** | **Cmin** | **AIC** | **BCC** |
| --- | --- | --- | --- |
| 36 | 753.599 | 825.599 | 871.885 |

**Final pollinator model (Fig. 3 D)**

| **Parameters** | **Cmin** | **AIC** | **BCC** |
| --- | --- | --- | --- |
| 22 | 768.347 | 812.347 | 840.633 |

**Initial Model:**

Pollination Benefit

e5

Flower Abundance

Flower Het.

Pollinator Diversity

Div*Het Interaction

Pollinator Composition 1

Pollinator Composition 2

Pollinator Composition 3

e7

e1

e2

e3

e4

e6
