# Supplementary material for: Resource Heterogeneity Moderates the Biodiversity-Function Relationship in Real World Ecosystems
Source: PLoS Biol. 2008 May 20;6(5):e122. doi: 10.1371/journal.pbio.0060122 (PMC2386840; doi:10.1371/journal.pbio.0060122)
Supplement: Supplementary file 9 [file pbio.0060122.st001.doc]

**Table S1: PCA statistics. 12 factors that explained all of the variance in heterogeneity of the original 12 soil parameters. Only factors 1-5 (in bold) were included in further analyses, as they had eigenvalues greater than 1 (i.e., the factor explained more variance than any one of the soil parameters). Heterogeneity of the soil variables was defined as the within-site coefficient of variation in the value/concentration of the variable.**

| **Factor number** | **Eigenvalue** | **% Total**  **variance** | **Cumulative**  **eigenvalue** | **Cumulative**  **% variance** |
| --- | --- | --- | --- | --- |
| **1** | **3.294631** | **27.45526** | **3.29463** | **27.4553** |
| **2** | **2.210440** | **18.42034** | **5.50507** | **45.8756** |
| **3** | **1.724824** | **14.37353** | **7.22990** | **60.2491** |
| **4** | **1.432895** | **11.94079** | **8.66279** | **72.1899** |
| **5** | **1.048067** | **8.73389** | **9.71086** | **80.9238** |
| 6 | 0.687185 | 5.72654 | 10.39804 | 86.6504 |
| 7 | 0.669933 | 5.58278 | 11.06798 | 92.2331 |
| 8 | 0.444657 | 3.70547 | 11.51263 | 95.9386 |
| 9 | 0.370145 | 3.08454 | 11.88278 | 99.0231 |
| 10 | 0.087127 | 0.72606 | 11.96990 | 99.7492 |
| 11 | 0.022787 | 0.18989 | 11.99269 | 99.9391 |
| 12 | 0.007310 | 0.06091 | 12.00000 | 100.0000 |
