# Supplementary material for: Resource Heterogeneity Moderates the Biodiversity-Function Relationship in Real World Ecosystems
Source: PLoS Biol. 2008 May 20;6(5):e122. doi: 10.1371/journal.pbio.0060122 (PMC2386840; doi:10.1371/journal.pbio.0060122)
Supplement: Supplementary file 10 [file pbio.0060122.st002.doc]

**Table S2: PCA factor loadings. Correlation between the 5 PCA factors used in analyses and the original soil heterogeneity variables. Heterogeneity of the soil variables was defined as the within-site coefficient of variation (CV) in the value/concentration of the variable.**

| **Variable** | **Factor 1** | **Factor 2** | **Factor 3** | **Factor 4** | **Factor 5** |
| --- | --- | --- | --- | --- | --- |
| **CV(pH (H_2_O))** | -0.673946 | -0.010276 | -0.395666 | -0.114985 | 0.070111 |
| **CV(NO_3_)** | 0.083850 | 0.052005 | -0.608923 | 0.324529 | 0.680187 |
| **CV(NH_4_)** | -0.031314 | -0.857229 | 0.282664 | 0.313409 | -0.199787 |
| **CV(Nmin)** | -0.037389 | -0.892460 | 0.179104 | 0.266419 | 0.137947 |
| **CV(N)** | -0.786310 | -0.015380 | 0.505209 | -0.215345 | 0.222465 |
| **CV(C)** | -0.432143 | 0.243452 | 0.690046 | -0.234347 | 0.440702 |
| **CV(C:N ratio)** | -0.773682 | -0.397617 | -0.208118 | -0.121152 | -0.084274 |
| **CV(Ca)** | -0.693500 | -0.154505 | -0.418682 | 0.145306 | -0.027421 |
| **CV(K)** | -0.523453 | 0.075792 | 0.095347 | 0.484505 | 0.180250 |
| **CV(Mg)** | -0.596703 | 0.026154 | -0.295180 | -0.415063 | -0.205115 |
| **CV(Na)** | 0.203792 | -0.459922 | -0.177725 | -0.667821 | 0.062921 |
| **CV(P)** | 0.524283 | -0.465695 | -0.087705 | -0.393777 | 0.437571 |
