# Supplementary material for: Resource Heterogeneity Moderates the Biodiversity-Function Relationship in Real World Ecosystems
Source: PLoS Biol. 2008 May 20;6(5):e122. doi: 10.1371/journal.pbio.0060122 (PMC2386840; doi:10.1371/journal.pbio.0060122)
Supplement: Supplementary file 11 [file pbio.0060122.st003.doc]

**Table S3:** Correlation coefficient (*r*) and significance level (*p*) for Pearson correlations between soil parameters and significant soil heterogeneity factor 5.

|  | **pH (H_2_O)** | **NO_3_** | **NH_4_** | **Nmin** | **N** | **C** | **C:N ratio** | **Ca** | **K** | **Mg** | **Na** | **P** |
| --- | --- | --- | --- | --- | --- | --- | --- | --- | --- | --- | --- | --- |
| ***r*** | .3904 | .1505 | -.0895 | .0184 | -.5151 | -.5812 | -.6534 | .0810 | -.0100 | .4629 | -.2069 | .6079 |
| ***p*** | p=.210 | p=.641 | p=.782 | p=.955 | p=.087 | p=.047 | p=.021 | p=.802 | p=.975 | p=.130 | p=.519 | p=.036 |
