# Supplementary material for: Resource Heterogeneity Moderates the Biodiversity-Function Relationship in Real World Ecosystems
Source: PLoS Biol. 2008 May 20;6(5):e122. doi: 10.1371/journal.pbio.0060122 (PMC2386840; doi:10.1371/journal.pbio.0060122)
Supplement: Supplementary file 12 [file pbio.0060122.st004.doc]

**Table S4: PCA statistics.** 12 factors that explained all of the variance in the absolute values of the 12 soil parameters (i.e., not variability as in Tables S1 and S2). Only factors 1-4 (in bold) were included in further analyses, as they had eigenvalues greater than 1 (i.e., the factor explained more variance than any one of the soil parameters).

| **Factor number** | **Eigenvalue** | **% Total**  **variance** | **Cumulative**  **eigenvalue** | **Cumulative**  **% variance** |
| --- | --- | --- | --- | --- |
| **1** | **4.513576** | **37.61313** | **4.51358** | **37.6131** |
| **2** | **2.021481** | **16.84567** | **6.53506** | **54.4588** |
| **3** | **1.653362** | **13.77801** | **8.18842** | **68.2368** |
| **4** | **1.558421** | **12.98685** | **9.74684** | **81.2237** |
| 5 | 0.837959 | 6.98299 | 10.58480 | 88.2067 |
| 6 | 0.554984 | 4.62487 | 11.13978 | 92.8315 |
| 7 | 0.469333 | 3.91111 | 11.60912 | 96.7426 |
| 8 | 0.261408 | 2.17840 | 11.87052 | 98.9210 |
| 9 | 0.088922 | 0.74102 | 11.95945 | 99.6621 |
| 10 | 0.037927 | 0.31606 | 11.99737 | 99.9781 |
| 11 | 0.001882 | 0.01569 | 11.99926 | 99.9938 |
| 12 | 0.000744 | 0.00620 | 12.00000 | 100.0000 |
