# Supplementary material for: Resource Heterogeneity Moderates the Biodiversity-Function Relationship in Real World Ecosystems
Source: PLoS Biol. 2008 May 20;6(5):e122. doi: 10.1371/journal.pbio.0060122 (PMC2386840; doi:10.1371/journal.pbio.0060122)
Supplement: Supplementary file 13 [file pbio.0060122.st005.doc]

**Table S5: PCA factor loadings.** Correlation between the 4 PCA factors used in SEM analyses and the original soil variables.

| **Variable** | **Factor 1** | **Factor 2** | **Factor 3** | **Factor 4** |
| --- | --- | --- | --- | --- |
| pH (H2O) | 0.914468 | -0.013510 | -0.061760 | 0.041953 |
| NO3 | 0.452826 | -0.323251 | 0.384014 | 0.535364 |
| NH4 | 0.601412 | -0.449081 | -0.178362 | -0.552145 |
| Nmin | 0.729474 | -0.506839 | 0.068598 | -0.315550 |
| N | -0.551019 | -0.736764 | 0.057245 | 0.279442 |
| C | -0.611417 | -0.748733 | 0.180451 | 0.065063 |
| CN | -0.389071 | -0.357785 | 0.431301 | -0.525761 |
| Ca | 0.772261 | -0.245772 | -0.010219 | 0.128659 |
| K | 0.141837 | 0.273902 | 0.819518 | -0.278131 |
| Mg | 0.867384 | -0.201753 | -0.316961 | 0.127179 |
| Na | -0.468694 | -0.220543 | -0.427736 | 0.272155 |
| P | 0.401348 | 0.045245 | 0.537183 | 0.568078 |
