# Supplementary material for: Resource Heterogeneity Moderates the Biodiversity-Function Relationship in Real World Ecosystems
Source: PLoS Biol. 2008 May 20;6(5):e122. doi: 10.1371/journal.pbio.0060122 (PMC2386840; doi:10.1371/journal.pbio.0060122)
Supplement: Supplementary file 14 [file pbio.0060122.st006.doc]

##### Table S6: Total effects for final belowground plant model

|  | Soil Factor 4 | Soil Factor 2 | Soil Factor 1 | Plant composition 2 | Plant composition 1 | Plant diversity | Soil Het. | Div.*Het.  Interaction |
| --- | --- | --- | --- | --- | --- | --- | --- | --- |
| Plant composition 1 | .439 | .301 | .682 | .000 | -.888 | -.014 | -.215 | .000 |
| Plant diversity | -1.088 | -.745 | -1.691 | .000 | 2.202 | -.965 | .533 | .000 |
| Soil Het. | .157 | .108 | .245 | .000 | -.319 | -.005 | -.077 | .000 |
| Div.*Het.  Interaction | .000 | .000 | .000 | -2.789 | .000 | .000 | .000 | .000 |
| Belowground  Biomass | -106.59 | -12.93 | -181.00 | 58.925 | 38.197 | .605 | 257.24 | 59.005 |
