# Supplementary material for: Resource Heterogeneity Moderates the Biodiversity-Function Relationship in Real World Ecosystems
Source: PLoS Biol. 2008 May 20;6(5):e122. doi: 10.1371/journal.pbio.0060122 (PMC2386840; doi:10.1371/journal.pbio.0060122)
Supplement: Supplementary file 15 [file pbio.0060122.st007.doc]

##### Table S7: Standardized total effects for final belowground plant model

|  | Soil Factor 4 | Soil Factor 2 | Soil Factor 1 | Plant composition 2 | Plant composition 1 | Plant diversity | Soil Het. | Div.*Het.  Interaction |
| --- | --- | --- | --- | --- | --- | --- | --- | --- |
| Plant composition 1 | .456 | .313 | .709 | .000 | -.888 | -.061 | -.226 | .000 |
| Plant diversity | -.260 | -.178 | -.404 | .000 | .506 | -.965 | .129 | .000 |
| Soil Het. | .156 | .107 | .243 | .000 | -.304 | -.021 | -.077 | .000 |
| Div.*Het.  Interaction | .000 | .000 | .000 | -.420 | .000 | .000 | .000 | .000 |
| Belowground  Biomass | -.273 | -.033 | -.464 | .078 | .094 | .006 | .666 | .518 |
