# Supplementary material for: Resource Heterogeneity Moderates the Biodiversity-Function Relationship in Real World Ecosystems
Source: PLoS Biol. 2008 May 20;6(5):e122. doi: 10.1371/journal.pbio.0060122 (PMC2386840; doi:10.1371/journal.pbio.0060122)
Supplement: Supplementary file 17 [file pbio.0060122.st009.doc]

##### Table S9: Standardized total effects for final aboveground plant model

|  | Soil Factor 4 | Soil Factor 2 | Soil Factor 1 | Plant composition 2 | Plant composition 1 | Plant diversity | Soil Het. |
| --- | --- | --- | --- | --- | --- | --- | --- |
| Plant composition 1 | .456 | .313 | .709 | .000 | -.888 | -.061 | -.226 |
| Plant diversity | -.260 | -.178 | -.404 | .000 | .506 | -.965 | .129 |
| Soil Het. | .156 | .107 | .243 | .000 | -.304 | -.021 | -.077 |
| Div.*Het.  Interaction | .000 | .000 | .000 | -.420 | .000 | .000 | .000 |
| Aboveground biomass | .398 | .273 | .618 | .000 | .098 | -.053 | -.197 |
