# Supplementary material for: Resource Heterogeneity Moderates the Biodiversity-Function Relationship in Real World Ecosystems
Source: PLoS Biol. 2008 May 20;6(5):e122. doi: 10.1371/journal.pbio.0060122 (PMC2386840; doi:10.1371/journal.pbio.0060122)
Supplement: Supplementary file 18 [file pbio.0060122.st010.doc]

**Table S10**: Total effects for final parasitoid model

|  | Host Abundance | Parasitoid composition 2 | Parasitoid diversity | Parasitoid composition 1 | Div*Het. interaction |
| --- | --- | --- | --- | --- | --- |
| Host Abundance | .000 | .000 | .000 | .000 | .000 |
| Parasitoid composition 2 | .000 | .000 | .000 | .000 | .000 |
| Parasitoid diversity | .002 | .000 | .000 | .000 | .000 |
| Parasitoid composition 1 | .001 | .000 | .543 | .000 | .000 |
| Div*Het. interaction | .000 | .000 | -.097 | -.179 | .000 |
| Proportion Parasitised | .000 | .073 | .091 | .123 | .082 |
