# Supplementary material for: Resource Heterogeneity Moderates the Biodiversity-Function Relationship in Real World Ecosystems
Source: PLoS Biol. 2008 May 20;6(5):e122. doi: 10.1371/journal.pbio.0060122 (PMC2386840; doi:10.1371/journal.pbio.0060122)
Supplement: Supplementary file 19 [file pbio.0060122.st011.doc]

##### Table S11: Standardized total effects for final parasitoid model

|  | Host Abundance | Parasitoid composition 2 | Parasitoid diversity | Parasitoid composition 1 | Div*Het. interaction |
| --- | --- | --- | --- | --- | --- |
| Host Abundance | .000 | .000 | .000 | .000 | .000 |
| Parasitoid composition 2 | .000 | .000 | .000 | .000 | .000 |
| Parasitoid diversity | .368 | .000 | .000 | .000 | .000 |
| Parasitoid composition 1 | .218 | .000 | .593 | .000 | .000 |
| Div*Het. interaction | -.088 | .000 | -.240 | -.405 | .000 |
| Proportion Parasitised | -.410 | .443 | .523 | .649 | .192 |
