# Supplementary material for: Resource Heterogeneity Moderates the Biodiversity-Function Relationship in Real World Ecosystems
Source: PLoS Biol. 2008 May 20;6(5):e122. doi: 10.1371/journal.pbio.0060122 (PMC2386840; doi:10.1371/journal.pbio.0060122)
Supplement: Supplementary file 20 [file pbio.0060122.st012.doc]

##### Table S12: Total effects for final pollinator model

|  | Flower Abundance | Pollinator Diversity | Pollinator Composition 3 | Div*Het Interaction |
| --- | --- | --- | --- | --- |
| Flower Het. | .366 | .000 | .000 | .000 |
| Pollinator Diversity | .000 | .000 | -2.951 | .000 |
| Pollinator Composition 3 | .000 | .000 | .000 | .000 |
| Div*Het Interaction | .000 | .000 | .000 | .000 |
| Pollination Benefit | .000 | .883 | -2.606 | .068 |
