# Supplementary material for: Resource Heterogeneity Moderates the Biodiversity-Function Relationship in Real World Ecosystems
Source: PLoS Biol. 2008 May 20;6(5):e122. doi: 10.1371/journal.pbio.0060122 (PMC2386840; doi:10.1371/journal.pbio.0060122)
Supplement: Supplementary file 21 [file pbio.0060122.st013.doc]

##### Table S13: Standardized total effects for final pollinator model

|  | Flower Abundance | Pollinator Diversity | Pollinator Composition 3 | Div*Het Interaction |
| --- | --- | --- | --- | --- |
| Flower Het. | .386 | .000 | .000 | .000 |
| Pollinator Diversity | .000 | .000 | -.582 | .000 |
| Pollinator Composition 3 | .000 | .000 | .000 | .000 |
| Div*Het Interaction | .000 | .000 | .000 | .000 |
| Pollination Benefit | .000 | .327 | -.190 | .553 |
